# Quality of life after volar locked plating: a 10-year follow-up study of patients with intra-articular distal radius fractures

**DOI:** 10.1186/1471-2474-15-250

**Published:** 2014-07-24

**Authors:** Paul Ruckenstuhl, Gerwin A Bernhardt, Patrick Sadoghi, Mathias Glehr, Lukas A Holzer, Andreas Leithner, Matthias Wolf, Gerald Gruber

**Affiliations:** 1 Department of Orthopedic Surgery, Medical University of Graz, Auenbruggerplatz 5-7, Graz 8036, Austria; 2 Department of Surgery, District Hospital of Weiz, Franz-Pichler-Straße 85, Weiz 8160, Austria

**Keywords:** Distal radius fractures, Health related quality of life, Volar plate fixation

## Abstract

**Background:**

This study aimed to present functional results and patient’s health related quality of life (HRQOL) data ten years after volar locked plate fixation (VPF) of unstable intra-articular distal radial fractures (DRF).

**Methods:**

Thirty-nine patients with a mean age of sixty-one years were operatively treated with VPF after intra-articular distal radial fractures. They were evaluated two, six, and ten years postoperatively according to the Gartland and Werley score. For subjective evaluation the Short Form 36 (SF-36) and the Disability of Arm, Shoulder and Hand (DASH) questionnaires were adopted.

**Results:**

Overall, wrist function did not differ significantly two, six and ten years after the operation. Over 90% patients achieved “good” or “excellent” results ten years after surgery according to the Gartland and Werley score. Ten years postoperatively the results of the SF 36 did not differ significantly from the two- and six-year follow-up. Overall findings from the SF-36 did not differ significantly from the data of Austrian and American norm populations. Only in the subscale of mental health (MH) the ten-year follow-up did show significantly poorer results (p = 0.045) compared to the Austrian norm population. The median DASH scores did not show significant differences during the ten-year follow-up period.

**Conclusion:**

The ten-year results of this single-center study suggest that operative treatment of intra-articular DRF with volar locked plates is a useful and satisfactory therapy option, both in terms of function and HRQOL.

## Background

Distal radius fractures (DRF) are the most common fractures in humans. Although most DRF can be treated non-operatively, unstable and intra-articular DRF need surgical treatment [1,2]. The objective for successful operative treatment is exact anatomic reduction with restoration of the initial articular surface as well as joint stability. It is reported that with improper restoration, these factors correlate with the development of posttraumatic arthritis and poor functional outcome [3-6]. Though many different methods have been proposed to fulfill these requirements, in recent years, a trend towards volar plate fixation (VPF) has developed [7-9]. VPF is safe and easy to perform, has low complication rates and provides excellent results in both the immediate postoperative period and in the long term [5,10,11].

While most orthopedic studies focus on special outcome parameters like postoperative pain, measures of impairment and abnormalities involving appearance, range of motion, loss of reduction, postoperative arthritis and others [12], several authors have recently looked at health related quality of life (HRQOL) after surgery [10,13]. Currently, there are a number of validated health assessments that focus on general health and HRQOL and can be used for comparisons with the health status of the general population, but the most frequently used questionnaire at present is the short form 36 (SF-36) that focuses on HRQOL [14]. In recent years HRQOL outcome measures have become increasingly important not only from a patient’s or doctor’s perspective but also for health insurers [15]. Poor results in HRQOL often correlate with longer periods of absence from work and with impact on public health and increase of insurance costs. Although studies on orthopedic and trauma surgery have produced large quantities of HRQOL data in recent years, data on HRQOL after treatment of intra-articular DRF are limited [16]. This type of data is of special interest as they could help the surgeon decide whether or not surgery is indicated, especially in the elderly patient. In 2010 we published our data on HRQOL after operatively treated intra-articular DRF [10]. Now we present a follow-up study that re-evaluated our cohort of intra-articular DRF treated with VPF after ten years and compared the data with the last follow-up as well as with HRQOL data of the norm population. To the best of our knowledge, no other long term data covering 10 years or more have been published.

## Methods

The study design, patient recruitment and surgical technique were published in our previous cohort analysis [10]. The study protocol was approved by the local ethics committee (Ethics committee of the Medical University of Graz, Austria, Institutional Review Board Registry, IRB00002556). All patients gave written informed consent to participate in the study. The study adhered to the STROBE statement (Strenghening the reporting of observational studies in epidemiology; http://www.strobe-statement.org) and the recommendations were checked. Initially 102 consecutive adult patients were treated with a volar locked plating system (I.T.S. [Implant-Technologie-Systeme], Lassnitzhoehe, Austria). Only patients with intra-articular DRF (Type C according to AO-classification of fractures) were considered for inclusion in the study. All VPF were performed by the same team of four surgeons as previously described [10].

### Clinical follow-up examination

The patients had routine clinical follow-up at four and twelve weeks. Follow-up examinations at one, two, six and ten years postoperatively were performed prospectively using a standardized protocol. Examinations were performed by surgeons not involved in the initial care of the patients. Sensation, tenderness, and range of motion of the wrist and forearm were evaluated. The range of motion of both wrists was measured in three planes (extension-flexion, radial-ulnar deviation, pronation and supination) with a goniometer that was placed along the axis of rotation of the wrist joint. To detect loss of muscular strength, we also compared the power grip between injured and uninjured hand in three grades (1 = no difference, 2 = minor weakness, 3 = major weakness) to every examination. Pain upon examination was assessed with the visual analogue scale (VAS, range 0-10).

The clinical data were quantified with the scoring systems of Gartland and Werley [17].

### HRQOL and subjective outcome measurement

The subjective outcome was assessed with the Short Form 36 (SF-36) and the Disabilities of the Arm, Shoulder and Hand (DASH) questionnaires [14,18]. At the two-, six- and ten-year visits, both questionnaires for patient-related outcome assessment were completed prior to the clinical examination. The DASH outcome measure consists of thirty self-reported questions designed to detect loss of function and symptoms in people with disorders of the upper extremity. The results are scaled from 0 to 100, with lower numbers indicating greater disability.

The SF-36 is a patient-reported questionnaire to survey health status (HS) and HRQOL. The score enables individuals to describe their health status from their own perspective. The SF-36 scores were compared with United States and Austrian norm populations [10].

The SF-36 questionnaire assesses eight domains including physical function (PF), role physical (RP), bodily pain (BP), vitality (VT), general health (GH), social function (SF), role emotional (RE) and mental health (MH) and two summary scores, mental component summary (MCS) and physical component summary (PCS). On its 100-point scale, high scores equate to good health, and low scores equate to poor health.

### Statistics

Parametrically distributed data are described as the mean and the standard deviation. Non-parametrically distributed data are given as the 95% confidence interval or the median and the range.

Student’s *T*-test, the Wilcoxon test, Friedman test and the analysis of variance (ANOVA) were used when appropriate. Follow-up comparisons were performed by post hoc testing with a Bonferroni-correction. Correlations were determined with Pearson correlations for parametric data and Spearman correlations for nonparametric data. All tests were two-sided with a significance-level of p < 0.05.

## Results

### Objective follow-up

**Figure 1:**
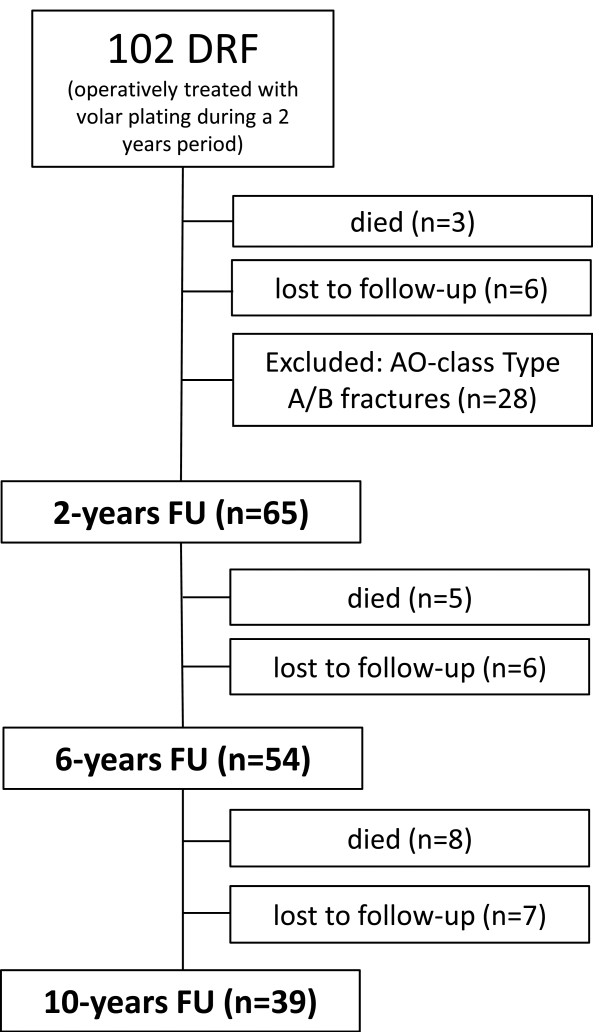
Development of the study group during the ten-year follow-up period.

The study group at the 10-year follow-up consisted of 39 patients, 24 which were women (62%) with a mean age of 61 ± 8 years (ranging from 28 to 87 years) at time of final follow-up. In 15 (38%) patients the dominant hand was involved. Patient’s flow-chart is illustrated schematically in Figure 1.

At final follow-up 3 patients (8%) were classified as typ-C1, 25 (64%) as type-C2 and 11 (28%) as type-C3 fractures. Overall there were no statistically significant differences in terms of wrist motion between the two-, six- and ten-year follow up examinations (Table 1).

**Table 1:** Wrist motion at two (FU-2y), six (FU-6y) and ten (FU-10y) years follow-up

***(mean ± SD)***	**FU-2y**	**FU-6y**	**FU-10y**
**Extension**	74 ± 10	62 ± 12	57 ± 14
**Flexion**	68 ± 15	56 ± 11	52 ± 13
**Radial deviation**	25 ± 6	26 ± 10	28 ± 6
**Ulnar deviation**	26 ± 8	33 ± 10	41 ± 9
**Supination**	84 ± 9	74 ± 11	75 ± 10
**Pronation**	86 ± 5	85 ± 9	85 ± 8

According to the Gartland and Werley score, there were no significant changes between the six- and ten-year follow-ups, though there was a decrease in function over time. Nonetheless, thirty-four patients (90%) achieved good or excellent results at ten-year follow-up. Table 2 shows the results of patients who were available for all three follow-up visits (n = 37). There were no significant differences between male and female patients.

**Table 2:** Results of the Gartland and Werley score at two (FU-2y), six (FU-6y) and ten (FU-10y) years follow-up

***n(%)***	**FU-2y**	**FU-6y**	**FU-10y**
**G1 (excellent)**	30 (81.1%)	28 (75.7%)	25 (67.6%)
**G2 (good)**	6 (16.2%)	9 (24.3%)	9 (24.3%)
**G3 (moderate)**	1 (2.7%)	0 (0.0%)	3 (8.1%)
**G4 (poor)**	0 (0.0%)	0 (0.0%)	0 (0.0%)

### Subjective follow-up

**Figure 2:**
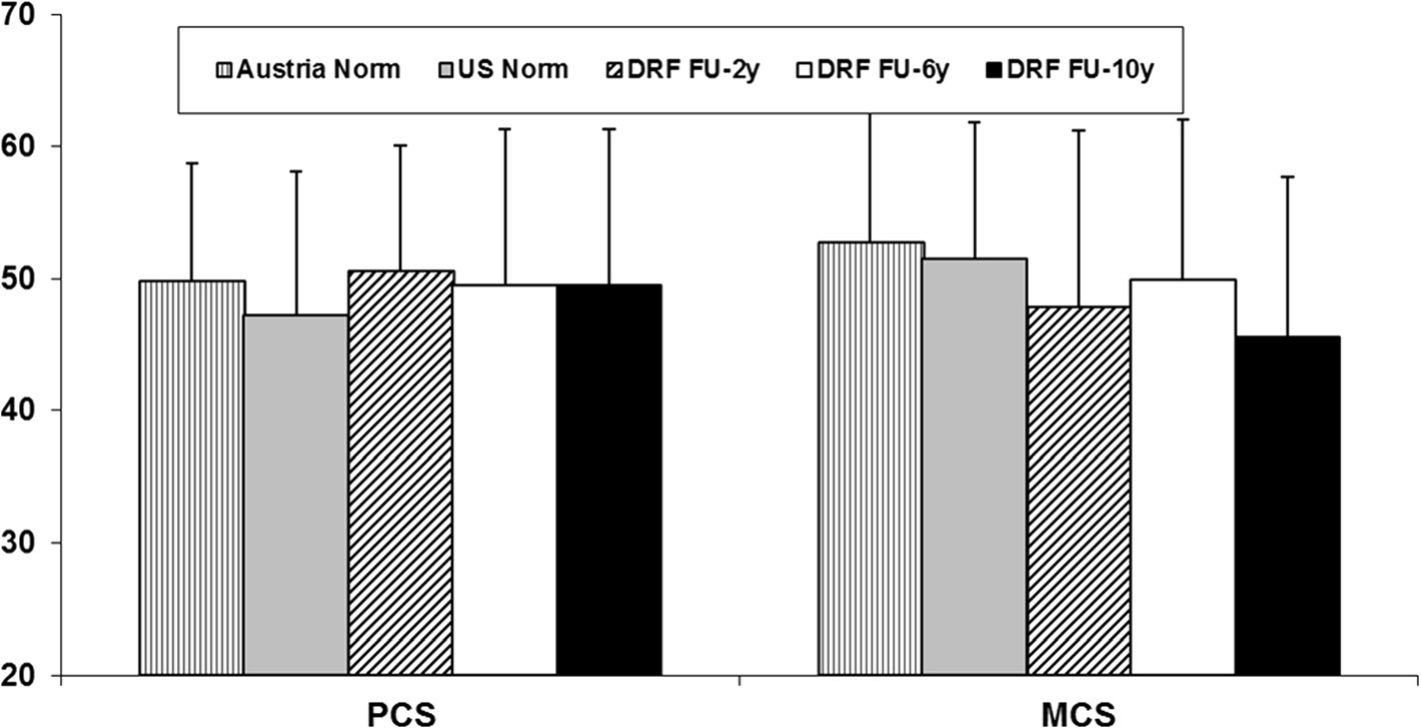
Results of the SF-36 summary scores at the two (FU-2y), six (FU-6y) and ten-year (FU-10y) follow-up including U.S. and Austrian population norms.

**Figure 3:**
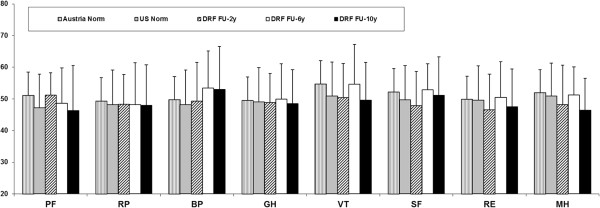
Results of the eight SF-36 sub scales at the two (FU-2y), six (FU-6y) and ten-year (FU-10y) follow-up including U.S. and Austrian population norms.

The overall results for the DASH score did not show significant differences within the follow-up period. The results deteriorated from two to six years after surgery, but improved between six and ten years almost to the two-year postsurgical level.The results of the SF-36 at ten-year follow-up for PCS and MCS did not show any significant changes compared to the follow-ups two and six years postoperatively. There were no significant differences in the PCS and MCS compared with the norm population data. In the MH (p = 0.045) subscale we found a general decrease comparing the ten-year follow-up results and the data of the Austrian norm population. Moreover, we found a significant decrease in the VT subscale from the six- to the ten-year follow-up. There was no significant difference between the ten-year follow-up examination and the US norm population (Figures 2 and 3).

There was a positive correlation between the MCS of the SF-36 and the DASH score results showing that patients with better DASH scores had better results in the MCS. This finding correlated with our previous investigations [10].

The median VAS was zero (range 0-5). At the time of the examination, 80% of all patients were completely free of pain.

The evaluation of muscular restriction showed positive results for ten patients (26%). Of these patients two (5%) presented a low and eight (21%) presented a clear level of weakness compared to the uninjured hand.

Out of the initial study group, four patients had a complication in the immediate post-operative period as described earlier [10]; there were no long term complications during the ten-year follow-up period.

In one of the patients with a complication the plate was removed; in two other patients the plating system was removed at the patient’s request.

## Discussion

The aim of this recent follow-up study was to re-evaluate our cohort of DRF treated with VPF after ten years and to compare the data with the last follow-up as well as with HRQOL data of the norm population. Today, a comprehensive evaluation after surgically treated DRF requires reliable, validated measures of functional and subjective outcomes. It is reported that with improper restoration of DRF patients very likely develope posttraumatic arthritis and poor functional outcome, on the other hand functional outcome and radiographic parameters do not necessarily correlate especially in the elderly and might not influence HRQOL [3-6,19,20]. We aimed to evaluate and compare clinical and subjective results after VPF of intra-articular DRF over a ten-year follow-up period. We found that ten years after VPF of intra-articular DRF, patients had good HRQOL compared to norm populations. Good long-term clinical results of operatively treated DRF with varying methods have already been reported [11,21-23]. The results after VPF are especially promising and seem to be superior to other procedures [7,8]. There is sufficient data to indicate that VPF has become more popular in recent years. VPF might move towards a status of gold standard method in the treatment of DRF, however data are lacking to show the unique superiority in comparison to other operation methods [4,5,7,24].

However, in the age of evidence-based medicine, health technology assessment (HTA) mandates the full and explicit evaluation not only of efficacy and safety but also of every other aspect that impacts on society (e.g. economic aspects) [15]. Furthermore, patients are demanding effective treatments that also yield the maximum HRQOL postoperatively. As a decrease in HRQOL after trauma is a major reason why patients seek surgical treatment, the patient has high expectations for resuming normal activities and to returning to work after surgery. The soon return to work is also an interest shared by health insurers and ultimately by the general public as well. It has become increasingly clear that priority should be given not only to clinical outcomes but also to the patient’s perception of HRQOL [10,16,25]. HRQOL is not measured directly but rather indirectly with measurement scales derived from questionnaires. We chose the SF-36 over other available instruments based on its widespread use in orthopedics and the strong evidence of validity [14]. The SF-36 is considered an attractive method for assessing HRQOL because of its brevity, rigorous psychometric development, and patient acceptance [14]. In terms of DRF very few studies included HRQOL outcomes as study end points [16] and there are no such data for VPF of intra-articular DRFs in the long term.

In the present study, the SF-36 was administered for self-completion by patients two, six and ten years postoperatively. The final follow-up results of PCS and MCS in our study did not show any statistically significant changes compared to the follow-ups at two and six years postoperatively. The results of the SF-36 questionnaire at the two-, six- and ten-year follow-ups compared with sex- and age-matched norms for the United States population and with data of an Austrian control group showed no significant changes in the PCS and MCS but there was a significant difference for the MH subscale (p = 0.045). In this subscale we found deterioration for our ten-year follow-up results compared to the Austrian norm, but no difference in comparison with the US norm population. The reason for this might ultimately be found in differences between the two health care systems. There was a decrease over time for the VT subscale that can be explained by the aging of our cohort. We found a positive correlation between VT and age but it may be assumed that general vitality will tend to deteriorate with increasing age. There were no significant differences in any other subscale of the SF-36 compared to the follow-up six years after surgery. There was a significant positive correlation between the MCS of the SF-36 and DASH scores, showing that patients with better DASH scores had better MCS results.

The overall results of the DASH score did not show significant differences within the follow-up period. However, with a median score of 0.8 (IQR 25-75; 0.0-30) at the ten-year follow-up the DASH score improved from 1.7 (IQR 25-75) at the six-year follow-up and almost reached its level for two years after the surgery, reflecting a low degree of upper extremity disability and symptoms. Similar results have been described in the literature [22,26]. Care must be taken that there might be a floor effect with the use of scoring systems that underestimates worse results. Upmost attention must be given when comparing DASH scores as they usually have a nonparametric distribution, due to the construction of the score. It is difficult to compare studies using the DASH as there are so few studies with a ten-year follow-up period.

The DASH score results and the Gartland and Werley score correlated over the ten-year period, showing that patients with higher DASH scores presented poorer functional outcomes.

Over the ten-year long study period we found that both the SF-36 and the DASH score were easy to process although it was difficult to motivate patients to participate in the study ten years after the operation. Fernandez et al. [27] encountered similar difficulties and concluded that the SF-36 and the DASH questionnaires are too long and too complicated to administer, in spite of their general acceptance as described above. Abramo and his group argue that the DASH requires substantial administrative work to ensure an acceptable frequency of replies [26].

In a recent systematic review, Van Son et al. [16] evaluated twenty-six studies dealing with HRQOL or health status (HS). The majority of studies had low methodological quality [16]. Van Son et al. report that representative statements regarding the HRQOL after DRF can be made by considering just the three domains of physical, psychological and social function in a balanced way. They [16] concluded that with the inconclusive results of mostly low-quality studies, there is a need for high-quality prospective follow-up studies measuring HRQOL and pointed out an information gap for HRQOL outcomes after DRF.

As a part of the SF-36 evaluation we detected a correlation between HRQOL and lifestyle. Patients with a history of smoking and alcohol consumption were associated with a lower HRQOL. These findings correspond to the result of Bhandari et al. [13] who evaluated HRQOL of patients with unstable ankle fractures.

It has to be considered that lifestyle habits influence the HRQOL independent of the injury and treatment. In any case, the identification of modifiable predictors of patient HRQOL could help in the choice of surgical approach and treatment [13].

The primary limitation of the present study is it’s rather small sample size, although adequate to detect moderate changes over time it may still limit the precision of our estimates. Due to demographic evolution, twenty-six patients died during the follow-up period, leaving a group of thirty-nine patients at the ten-year follow-up. Another limitation of our study is that we did not include non-operatively treated patients with intra-articular DRF as a control group. However, it would have been difficult to compare such groups as unstable and more complex fractures need surgical treatment. A drawback of our study is that we did not assess preoperative and immediate postoperative SF-36 data so that we were not able to compare these data with our long-term follow-up results. The two- and six-year assessments were made at the hospital outpatient clinic, while some of the final follow-up evaluations were scheduled as home visits to accommodate patients and to minimize drop-out. This course of action might have created bias in the patient’s subjective outcome. Although we used sex- and age-matched Austrian and US normative SF-36 data, these are historical controls that may not be generalizable to our patients. As the age- and gender-matched controls did not exclude people with chronic conditions, the findings represent a cross section of the population and not the health status of “normal” individuals. However, it can be estimated that chronic disorders and comorbidities equally influence the general outcome of SF-36 in both the DRF study group and the norm population. Furthermore there are general limitations that arise in studies that use questionnaires such as the SF-36: they do not account for the specific settings of the patients who have undergone surgical repair of the distal radius. Some authors argue that the SF-36 is an instrument for measurement of general HS rather than a HRQOL assessment tool [16]. For HRQOL assessment, the WHOQOL-Bref [28] would have been an alternative suitable instrument, although specific problem areas in DRF might have been missed, because of its generic nature. Ideally, the constructions of a valid and specific subjective DRF outcome measure would provide more information on HRQOL in these patients. To minimize and to account for this problem we also used the DASH questionnaire in our study as it describes patient-related outcome assessment including disabilities of the hand [18]. Several studies have demonstrated usefulness of the DASH questionnaire [10,24,25,29] although it mainly assesses physical functioning and only touches on psychological and social functioning, which fails to meet the requirement of multidimensionality of HS and HRQOL as defined by the WHOQOL group [30].

In addition to the SF-36 and the DASH, we evaluated the Gartland and Werley score. In the most recent follow-up we were not able to assess Castaing score as we did in our previous follow-ups, as it would have been unethical to recall the patients for another radiological follow-up visit ten years after the initial operation. Since HRQOL and clinical results did not change significantly over time, it seems unlikely that x-ray studies would have provided relevant new information.

## Conclusion

For the first time, we provide long-term results for a VPF cohort up to ten years after intra-articular DRF. The satisfactory results of the present study confirm and describe what has already been known regarding functional outcome after volar plating of AO type C distal radius fractures in the short- and mid-term. The stable results of the subjective outcome measurement tools (SF-36, DASH) underline the safety and efficiency of VPF of articular DRF. Most of our patients achieved good clinical and HRQOL results in the long term. Based on our ten year results, further significant changes regarding HRQOL in comparison with norm populations are very unlikely.

Still, more data is needed to substantiate our results. Ideally, data including HRQOL after DRF for patients who have undergone both conservative as well as surgical treatment should be entered prospectively in multinational databases and evaluated on a regular basis [31].

## Abbreviations

DASH: Disability of Arm, Shoulder and Hand; DRF: Distal radius fracture; FU: Follow up; GH: General health; HRQOL: Health related quality of life; HS: Health status; MCS: Mental component summary; MH: Mental health; PCS: Physical component summary; PF: Physical functioning; VPF: Volar locked plate fixation; RE: Role emotional; RP: Role physical; BP: Bodily pain; SD: Standard deviation; SF-36: Short Form 36; SF: Social functioning; VAS: Visual analoge scale; VT: Vitality.

## Competing interests

The authors declare that they have no competing interests. No external funding was received for this study.

## Authors’ contributions

PR and GAB conducted the study, analyzed and interpreted the data and wrote the manuscript. PS participated in data analyzing, statistics and helped to draft the manuscript. MG, LAH, AL, MW helped in data acquisition including clinical work up and revised the manuscript critically. GG and GAB designed the study, wrote the study protocol revised the manuscript critically. All authors gave final approval of the manuscript before submission.

## Pre-publication history

The pre-publication history for this paper can be accessed here:

http://www.biomedcentral.com/1471-2474/15/250/prepub
